# Transconjunctival Sutureless 23-gauge Vitrectomy for Vitreoretinal Diseases: Outcome of 30 Consecutive Cases

**DOI:** 10.4103/0974-9233.51983

**Published:** 2008

**Authors:** Ashraf M. El-Batarny

**Affiliations:** From the Department of Ophthalmology, Magrabi Eye and Ear Hospital, Muscat, Sultanate of Oman

**Keywords:** 23-gauge vitrectomy, transconjunctival sutureless vitrectomy, vitreoretinal surgery

## Abstract

**Background:**

To describe the initial experience, effectiveness, and safety profile of 23-gauge instrumentation for a variety of vitreoretinal conditions.

**Methods:**

A retrospective review of 30 consecutive 23-gauge vitrectomy cases done by a single vitreoretinal surgeon for various posterior segment conditions was done. All surgeries were performed using the two-step 23-gauge system developed by Dutch Ophthalmic Research Center (DORC). All patients had at least 3-month follow-up. Main outcome measures included surgical success, visual acuity, intraocular pressure, and operative complications.

**Results:**

Mean follow-up was 7.7 months (range 3–12 months). Indications for surgery included rhegmatogenous retinal detachment (n=8), nonclearing vitreous hemorrhage (n=6), tractional retinal detachment (n=5), macular hole (n=5), epiretinal membrane (n=3), retained lens fragments (n=2) and endophthalmitis (n=1). Gas tamponade was used in 18 eyes (60%) and silicone oil in six eyes (20%). Mean overall preoperative visual acuity was 20/1053 and final acuity was 20/78 (*P* = 0.001). Mean intraocular pressure after 6 hours was 15.1mmHg (range 4-25 mmHg) and on postoperative day one was 14.5 mmHg (range 2-21 mmHg). Four eyes (13.3%) required suturing of sclerotomy intraoperatively. Conversion to 20-gauge was done in one eye (3.3%). Hypotony was reported in one eye (3.3%) postoperatively. Subconjunctival silicone oil reported in one eye (3.3%). There were no postoperative complications of endophthalmitis, retinal or choroidal detachment.

**Conclusion:**

23-gauge transconjunctival sutureless vitrectomy was effective in the management of wide variety of vitreoretinal surgical indications. The safety profile compared favorably with published rates for 25-gauge systems.

Modern vitrectomy surgery has classically been performed with 20-gauge instruments. However, advancement in techniques and instrumentation led to the introduction of 25-gauge transconjunctival sutureless vitrectomy. One of the most innovative vitreoretinal surgery techniques introduced in recent years is transconjunctival sutureless vitrectomy developed by Fujii et al.^1^,^2^

Sutureless posterior segment surgery provides numerous potential advantages over traditional 20-gauge vitrectomy, including faster wound healing, diminished conjunctival scarring, improved patient comfort, decreased postoperative inflammation, and reduced postoperative astigmatism.^3^–^11^ eliminating suturing may also shorten surgical opening and closing times.^12^

However, compared with traditional 20-gauge systems, postoperative rates of wound leakage, hypotony, and choroidal detachment may be higher in 25 gauge surgery.^12^–^14^

One of the most frequent objections is that the 25-gauge instruments are too flexible for many of the complicated tasks performed on the retina and vitreous body.^15^ Several reports have documented intraoperative and postoperative retinal tears and detachments, potentially as a result of lack of adequate peripheral vitrectomy with the more flexible instruments and subsequent excessive vitreoretinal traction at sclerotomy sites.^1^,^16^ A new approach using a 23-gauge system may eliminate some of these disadvantages. Twenty three-gauge vitrectomy involves making transconjunctival sutureless self sealing angled sclerotomies.^15^ Twenty-three gauge instruments are larger and thought to have improved rigidity over 25-gauge instruments, thus allowing greater rotation of the eye and the ability to perform a more complete peripheral vitrectomy. A minimal residual peripheral vitreous skirt may be associated with a decreased risk of postoperative retinal detachment.^16^ Also, with smaller incisions than standard 20-gauge vitrectomy; postoperative patient comfort and recovery are considered to be improved. However, as with 25-gauge vitrectomy, there are theoretical concerns of postoperative endophthalmitis and hypotony from unsutured sclerotomies with 23-gauge vitrectomy.^17^ An advantage of this instrument system is that it utilizes tunneled sclerotomy through the use of a slanted microvitreoretinal blade followed by a blunt trocar, which may provide an improved self-sealing incision.^18^ This study examines the safety and efficacy of 23-gauge vitrectomy in a consecutive series of cases.

## Methods

Approval was obtained from the ethics review board of Magrabi hospital for this study. After discussing the nature of surgery with the patients including the potential risks and complications, all subjects signed a written informed consent document before surgery. A retrospective review of 30 consecutive patients who underwent 23-gauge transconjunctival sutureless vitrectomy by a single surgeon was conducted. Indications for surgery included rhegmatogenous retinal detachment (RRD) (n =8), nonclearing vitreous hemorrhage (NCVH) (n =6), tractional retinal detachment (TRD) (n =5), macular hole (n=5), epiretinal membrane (ERM) (n=3), retained lens fragments (RLF) (n=2) and endophthalmitis (n =1). Eyes with a minimum of 3-month follow-up were included in this study. Eyes that had undergone prior vitrectomy surgery were excluded from this study.

Preoperative data obtained from patients charts included age, gender, date and indication of surgery, preoperative best corrected visual acuity (BCVA), intraocular pressure (IOP) and lens status.

Intraoperative data included success to achieve surgical goals in each case, development of intraoperative retinal tears, conversion to 20-gauge instrumentation, leakage, intraoperative suturing of sclerotomies, internal tamponade and any intraoperative complication.

Postoperative data included Postoperative intraocular pressure measured 6 hours, one day, and one week, BCVA at last visit, postoperative anatomical success, lens status and complications.

### Surgical Technique

**Figure 1:**
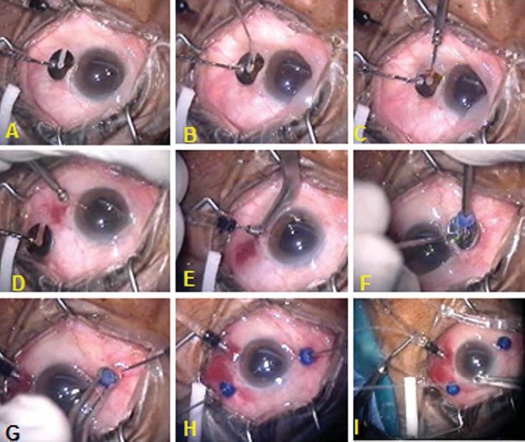
Transconjunctival insertion of 23- gauge cannulas. **A,** A toothed pressure plate is used to displace the conjunctiva and stabilize the globe. The center of the plate is 3.5 mm from the limbus. **B,** A 45° angled 23-gauge microvitreoretinal blade is inserted in the conjunctiva and sclera at 20° angle, parallel to the limbus. **C,** Insertion of blunt trocar and cannula through the scleral incision in the center of pressure plate. **D,** Removal of pressure plate. **E,** the blunt trocar is removed and a 23-gauge infusion cannula is inserted. **F,** Introduction of upper nasal cannula with a valve fitting on its port. **G,** The cannula is fixed with a special forceps and the trocar is removed. **H,** Twenty three gauge cutter and light probe are inserted in upper ports through the valves. **I,** One of the Chandelier twin light fiberoptics is inserted through 27- gauge needle puncture into the 23- gauge infusion cannula to create an illuminated infusion cannula for bimanual maneuvers.

Each patient underwent a 23-gauge transconjunctival sutureless vitrectomy using the Dutch Ophthalmic Research Center two step-system (DORC, Zuidland, The Netherlands) (Fig 1). The conjunctiva was displaced with a pressure plate with a central opening 3.5 mm from the edge. Angled incisions were made in the conjunctiva and sclera through the pars plana with a 23-gauge 45° angled microvitreoretinal (MVR) blade in the inferotemporal, superonasal, and superotemporal quadrants parallel to the limbus. The blade was inserted at an angle of approximately 20∞ to the sclera, 3.5 mm posterior to the limbus through the central opening of the pressure plate. While keeping the pressure plate in place, the blunt microtrocars were inserted through these wounds and the infusion cannula was placed in the inferotemporal quadrant, while the superonasal and superotemporal cannulas were used for the retinal instrumentation. The Accurus Vitrector (Alcon Laboratories, Inc., Fort Worth, TX) was used for all surgical procedures.

A core vitrectomy was then performed and triamcinolone acetonide was used to ensure that the posterior hyaloid was elevated and removed in every case. Trypan blue was used to assist epiretinal and internal limiting membrane stripping in epiretinal membrane and in macular hole cases using 23-gauge microforceps. In cases of diabetic vitrectomy, all cases were given 1.25 mg bevacizumab intravitreal injection 5-7 days before surgery to assist in controlling intraoperative bleeding. Intraoperatively, endolaser treatment was applied with a curved 23-gauge laser probe (Nidek, Japan). For tractional retinal detachments, membrane dissection was performed primarily with the 23-gauge vitrectomy probe and hemostasis was achieved using 23-gauge endodiathermy. Whenever bimanual dissection was needed, the 27gauge Eckardt chandelier twin lights (DORC). were inserted in the upper quadrant or one of the 27-gauge twin lights was inserted into the infusion cannula through 27 gauge needle oblique puncture creating an artificial illuminated infusion (Figure 1, I). Rhegmatogenous retinal detachments were repaired using a posterior retinotomy created with endodiathermy and an air-fluid exchange using a backflush brush needle. Focal endolaser treatment was applied around the retinotomy site, retinal breaks, and any areas of suspicious peripheral retinal pathology. In all cases, scleral depression was performed at the conclusion of the case by using the binocular indirect ophthalmomicroscope (BIOM) wide angle viewing system (Oculus, Germany) to evaluate for retinal breaks. In case of gas tamponade, a full air-fluid exchange was performed. Both the superonasal and superotemporal sclerotomies were plugged and the infusion line was then clamped at a pressure of 30 mmHg. The superonasal cannula was removed and the conjunctiva repositioned to cover the sclerotomy site. Infusion line was then unclamped and the sclerotomy site was inspected for any leakage of air, which can be easily seen under the conjunctiva. The infusion line was clamped again and the superotemporal cannula was then removed similarly. After removal of the upper cannulas, the vitreous cavity was flushed with 20 mL 25% sulpher hexafluoride (SF6) gas through the pars plana and then the infusion is immediately clamped and removed in a similar way. In cases having silicone oil, after having air fluid exchange, silicone oil, 1000 centistokes was injected through the upper temporal cannula while the upper nasal one was plugged until the whole vitreous cavity was filled and silicone oil spilled out in the infusion cannula which was then clamped. The upper temporal cannula was then plugged and both upper cannulas were removed. The eye was tonized whenever needed by injection of silicone through the infusion cannula which was finally removed. If any of the ports demonstrated persistent leakage, the conjunctiva was opened over the sclerotomy site and 7-0 Vicryl sutures were placed in the wound and the overlying conjunctiva. The eye was treated with a moxifloxacin (Vigamox, Alcon Laboratories, Fort Worth, TX) eye drops during the post operative period.

Main outcome measures included BCVA, IOP, and both intraoperative and postoperative complications. Snellen's visual acuity was converted into logarithm of the minimum angle of resolution (logMAR) for statistical analysis. Hypotony was defined as an intraocular pressure less than 6 mmHg. Paired Student t-test was performed to compare the preoperative and postoperative results within the same group. The unpaired Student t-test was used to compare means between different subgroups.

## Results

Thirty eyes of 30 patients underwent 23-gauge vitrectomy. The mean age was 54 ±13 years. (Range 16-72 years). Fourteen patients were women. Seventeen eyes were phakic with varying degrees of cataract documented in ten eyes, 12 eyes were pseudophakic, and one eye was aphakic. Additional procedures included phaco-surgery in seven eyes, pars plana lensectomy in two eyes (16 and 18 years old patients), anterior chamber intraocular lens implantation in one eye and intravitreal injection of antibiotics in one eye. The mean follow up was 7.7 ± 2.1 months (range 3-12 months). Surgical goals were achieved in all cases.

The mean overall preoperative BCVA was 20/1053 ± 7.8 Snellen's lines (range hand motions – 20/67). At the final postoperative visit, the mean BCVA improved to 20/78 ± 2.5 lines (range 20/400 – 20/28) (P=0.001). (Table 1)

**Table 1:** Visual Acuity Changes in All Case Series and Subgroups

Subgroups by Diagnosis	Preoperative BCVA mean ± SD (Range)	Postoperative BCVA mean ± SD (Range)	*P* value
All patients (n=30)	20/1053± 7.8 (HM- 20/67)	20/78 ± 2.5 (20/400 - 20/28)	0.001

RRD (n=8)	20/2000 ± 7.2 (HM - 20/400)	20/6612 (20/133-20/40)	0.001

NCV H. (n=6)	20/1429 ± 7.5 (HM - 20/133)	20/8712.4 (20/200 -20/50)	0.011

TRD (n=5)	20/2000 ± 9.3 (HM - 20/200)	20/133 ± 1.1 (20/200 - 20/100)	0.045

MH (n=5)	20/385±4.5 (CF -20/133)	20/78 ± 4.1 (20/400 -20/40)	0.001

ERM (n=3)	20/167 ± 1.2 (20/200 -20/133)	20/63 ±0.8 (20/67- 20/50)	-

RLF (n=2)	20/167 ± 5.3(20/400-20/67)	20/39 ± 1.5 (20/50 - 20/28)	-

Endophthalmitis(n=1)	20/2000	20/100	-

BCVA = Snellen best corrected visual acuity; SD = standard deviation; HM = hand motion; CF counting fingers at 2 feet; RRD = rhegmatofenous retinal detachment; NCV H = vitreous hemorrhage; TRD = fractional retinal detachemnt; MH = macular hole; ERM = epiretinal membrane; RLF = retained lens fragments

Regarding the intraoperative complications, localized subconjunctival hemorrhage during insertion of the cannula was observed in five out of 30 cases (16.7%). One eye out of 30 eyes (3.3%) had iatrogenic tear which was related to dissection of preretinal membrane in TRD. No tears related to sclerotomy sites were reported. Conversion to 20-gauge vitrectomy was required in one case (3.3%) with retained lens fragments, where fragments of epi-nucleus were too hard to be removed with the cutter. In this case, only one port was converted to 20-gauge to accommodate the fragmatome tip. Leaking sclerotomies requiring suturing at the end of vitrectomy were reported in four cases (13.3%); three of which had silicone oil and in one case, no intraocular tamponade was used. Leaking sclerotomies were reported in 50% of silicone oil cases (three out of six eyes). Subconjunctival silicone oil was reported in one case. Hypotony was reported in one eye out of 30 eyes (3.3%). This case, which did not have silicone or gas tamponade, had severe hypotony with folds observed in the retina at the posterior pole requiring tonization with SF6 gas on the fourth postoperative day.

Postoperatively, no cases of retinal detachment, choroidal detachment or endophthalmitis were reported. Deterioration of cataract was observed in three eyes out of eight phakic eyes (37.5%), two cases underwent cataract extraction and intraocular lens implantation during follow up.

Mean overall preoperative intraocular pressure was 12.4 ± 3.5 mmHg (range 6-23 mmHg). Mean overall postoperative intraocular pressure six hours after surgery was 15.1 ± 5.5 mmHg (range 4-25 mmHg), on day one was 14.5 ± 4.6 (range 2-21 mmHg), and after one week was 13.7 ± 1.9 mmHg (range 10-18 mmHg) (Table 2). The difference between preoperative intraocular pressure and postoperative intraocular pressure at six hours was statistically significant (P=0.046). The differences of preoperative intraocular pressure and postoperative intraocular pressure at day one (P=0.086) and at one week (P=0.14) were not statistically insignificant. Table 2 compares the intraocular pressure means between subgroups according to intraocular tamponade at preoperative and different postoperative measurement points. The mean preoperative intraocular pressure in fluid filled eyes (n=6) was 13.8 ± 4.8 mmHg (range 10-23 mmHg). Six hours after surgery it was significantly reduced to 6.3 ± 1.6 mmHg (range 4-8 mmHg) (P=0.027). The difference thereafter was not statistically significant (P at day 1 = 0.11 and P at 1 week = 0.36).

**Table 2:** Intraocular Pressure Changes Before and After Surgery in Overall Cases and in Subgroups According to Intraocular Tamponade

IOP (mmHg) mean ± SD, (range) P value* Subgroups	Preoperative	6 hours	1 day	1 week
Allover (n=30)	12.4±3.5(6-23)	15.1 ±5.5 (4-25) P=0.046	14.5±4.6 (2-21) P=0.068	13.7±1.9 (10-18) P=0.14

Gas (n=18)	12.5±3.3(6-20)	18.3±3.4(14-25) P=0.001	19.9±3.1(12-21) P=0.001	14±1.7(12-18) P=0.15

Fluid (n=6)	13.8±4.8(10-23)	6.3±1.6(4-8) P=0.027	7.8±2.9(2-10) P=0.11	12.3±1.7(10-15) P=0.36

Silicone oil (n=6)	10.7±2.2(8-14)	14.2±2.3(10-17) P=0.09	13.7±2.4(10-16) P=0.12	14±2.5(12-17) P=0.06

IOP = intraocular pressure; SD = standard deviation;

* *P* value represents comparison between pre- and post-operative IOP at each measurement point.

The mean preoperative intraocular pressure in gas filled eyes was 12.5 ± 3.3 mmHg (range 6-20 mmHg). It became significantly higher at six hours (P=0.001) and one day (P=0.001) then it approached the preoperative value after one week (P=0.147).

**Figure 2:**
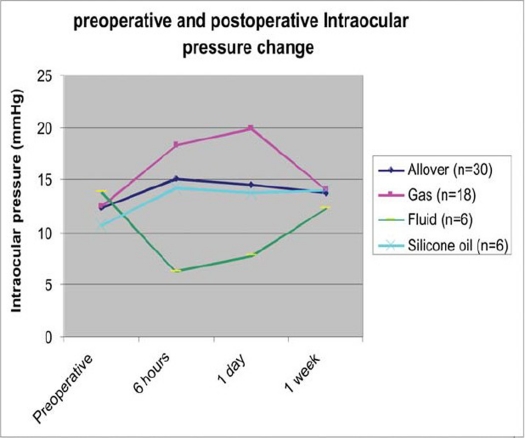
Illustration of the behavior of intraocular pressure (IOP) during the early postoperative period. In fluid filled eyes, IOP was lowermost among all groups, six hours and at day one after surgery. In gas filled eyes, IOP was highest among all groups' six hours and at day one after surgery. At one week, all groups approached the preoperative value.

In silicone filled eyes, no significant difference was found between the preoperative pressure and postoperative pressure at all measurement points (P at six hours = 0.09, P at day one=0.12 and P at one week= 0.06). Figure 2 describes the behavior of IOP in different subgroups in the early postoperative period. The mean intraocular pressure after surgery was lowest in cases where no tamponade was left in the eye compared to gas and silicone oil filled cases at six hours and on day one. Comparing the intraocular pressure means between fluid and gas filled eyes, the difference was significant at six hours (P=0.001), and on day one (P=0.001).but not after one week (P=0.088). After one week there was no significant difference between preoperative and postoperative IOP means in different subgroups.

In the eight eyes with rhegmatogenous retinal detachment, all eyes had macular detachment. One eye in a high myopic 16 years old patient had subluxated lens which underwent additional lensectomy. Four eyes have silicone oil tamponade and the other four had 25% SF6 gas. No additional buckle was used in any of the cases. All cases were successfully attached. The mean preoperative visual acuity was 20/2000 ± 7.2 Snellen's lines (range hand motions – 20/400) and mean postoperative visual acuity was 20/66± 2 lines (range 20/133-20/40) (P=0.001).

In six eyes with non clearing vitreous hemorrhage, etiology of the hemorrhage was proliferative diabetic retinopathy (PDR) (n=4), branch retinal vein occlusion (n=1) and penetrating trauma (n=1). The traumatic hemorrhage was associated with subluxated cataractous lens which underwent additional lensectomy (age 18 years old). The mean preoperative visual acuity was 20/1429 ± 7.5 (range hand motions – 20/133) which improved to 20/87 ± 2.4 lines (range 20/200-20/50) (P=0.011).

Five eyes had tractional retinal detachment due to PDR. The mean preoperative visual acuity was 20/2000 ± 9.3 lines (range hand motions – 20/200) which improved to 20/133 ± 1.1 lines (range 20/200-20/100) (P=0.045).

Five eyes had macular holes; four idiopathic and one myopic with detachment restricted to posterior pole. All received SF6 gas. Hole closure was achieved in all cases initially but in the myopic eye, the hole reopened five months postoperatively. The mean preoperative visual acuity was 20/385 ± 4.5 lines (range finger counting @ 2 feet – 20/133) which improved to 20/78 ± 4.1 lines (range 20/400-20/40) (P=0.001).

Three cases had epiretinal membranes, two idiopathic and one following buckle surgery. No tamponade was used in all cases. The mean preoperative visual acuity was 20/167 ± 1.2 lines (range 20/200-20/133) which improved to 20/63 ± 0.8 lines (range 20/67-20/50). Sample size was insufficient for proper statistical analysis.

Two eyes had retained lens fragments with associated intraocular inflammation. Secondary glaucoma was present in one case. Anterior chamber lens was implanted in one eye. In one eye conversion of one port to 20-gauge was required to use the fragmatome tip. The mean preoperative visual acuity was 20/167 ± 5.3 lines (range 20/400-20/67) which improved to 20/39 ± 1.5 lines (range 20/50-20/28). Sample size was insufficient for proper statistical analysis.

One eye of endophthalmitis following complicated phaco-surgery was included in the study. The vitrectomy cutter was used to remove inflammatory membranes in the anterior chamber and on the iris. Intravitreal antibiotic (vancomycin and ceftazidime) were injected in the vitreous cavity at the end of the procedure. No tamponade was used in this case. Vision improved from 20/2000 to 20/100.

## Discussion

No advance in the treatment of Vitreoretinal diseases has been as significant as the introduction of pars plana vitrectomy by Machemer in 1971.^19^ The original multifunctional probe provided endoillumination and removal of vitreous through one instrument inserted through 3.3 mm pars plana sclerotomy. In 1975, Dr Carl Wang developed his Ocutome^®^ 800 at set of 20 gauge instruments that became the first complete system for posterior vitrectomy.^20^ One of the most innovative Vitreoretinal surgery techniques introduced in recent years is transconjunctival sutureless 25-gauge microsurgical vitrectomy techniques developed by Fuji et al in 2002.^1^,^2^ The procedure has quickly found many advocates. It causes no surgical trauma to the conjunctiva, requires no scleral suture (and thus leaves no postoperative suture-related astigmatism), and entails a distinctly reduced rehabilitation time.^15^ Many Vitreoretinal surgeons, however, reject the method at all or accept it only for special indications. One of the most frequent objections is that the 25-gauge instruments are too flexible for many of the complicated tasks performed on the retina and vitreous body.^15^ After 25-gauge trocars (that were inserted perpendicular to the sclera) are removed, an open hole in the sclera is often clearly visualized and represents a direct opening from the vitreous to the outside of the eye, putting the eye at increased risk for endophthalmitis.^17^ In addition, openings in the sclera can be associated with wound leakage. Hypotony in the postoperative period can increase the risk for complications, including retinal and vitreous incarceration and suprachoroidal hemorrhage.^13^,^14^ In a review of 140 consecutive 25-gauge cases, Lakhanpal et al reported that 7.1% of eyes required a suture for adequate closure and 3.8% demonstrated postoperative choroidal detachment.^12^ A retrospective study of 71 eyes by Yanyali and associates demonstrated intraocular pressure between 6 mmHg and 10 mmHg in 16.9% of the eyes on the first postoperative day but normalization thereafter.^11^

A recent innovation has been the 23-gauge transconjunctival sutureless vitrectomy system introduced by Echardt 2005 which overcomes many of the shortcomings of the 25-gauge system.^15^ With the larger gauge, the instruments are less flexible allowing better manipulation of the eye with more complete vitrectomy.^17^

In the current series, there was a statistically significant improvement of vision with the 23-gauge system. Visual improvement was also noted in most of subgroups. The complications reported as development of iatrogenic tear during membrane dissection in TRD and reopening of macular hole in high myopic eye could be attributed to the underlying pathology rather than to 23-gauge surgical technique. No tears related to the sclerotomy trocars were reported. This could be explained by several factors. The tough instruments allow a more controlled peripheral vitrectomy. The possibility to exchange the cutter position between the trocars including the lower temporal infusion trocar whenever needed allowed more efficient peripheral vitrectomy. In addition, the 23-gauge cutter used allowed a cutting speed at 100-2500 cuts per minute (cpm) compared to 100-1500 cpm with 25-gauge probe. The cutter port is 50% closer to the probe tip compared to the 20-gauge cutter. These criteria of the 23-gauge cutting probe allow efficient vitreous shaving close to the retina without inducing tears. The recent innovation of the valves applied on the top of the trocars prevent the reflux of fluid out of the ports following extraction of instruments out of the eye, thus preventing vitreous incarceration into the trocars.

Intraoperative leakage requiring suturing was reported in four cases. Postoperative hypotony requiring tonization with gas injection was reported only in one fluid filled eye. Actually, several factors can contribute to postoperative leakage and hypotony. Creation of short scleral tunnel during insertion of the trocars can lead to leakage after removal of the trocars at the end of surgery. By using an incision angle of 45° to the scleral surface, a tunnel of 1.154 mm long will be created. By reducing the incision angle to 25-30°, a tunneled wound would be roughly 1.4mm long (if the scleral thickness 3.5 mm posterior to the limbus is 0.6 mm) which is more air tight.^18^,^20^ In the current series, the knife was inserted at approximately 20° angle trying to achieve as long as possible self sealed tunnels. The postoperative IOP was significantly lower in fluid filled eyes compared to gas filled eyes in the early postoperative period. This means that the type of tamponade can affect the tightness of the wound and that gas or air would be associated with less possibility of leakage and hypotony compared to fluid. Some surgeons now use a gas fill to plug 25-gauge sclerotomies.^18^ In the current series, the IOP was significantly higher than preoperative value at six hours and day one after surgery corresponding to the time of expansion of the gas bubble. Fine et al, reported that about 14.3% of cases had rise of intraocular pressure following 23-gauge vitrectomy due to intraocular gas bubble or topical steroid therapy.^18^

Three out of the four cases requiring intraoperative sutures were filled with silicone oil. This represents 50% of silicone filled eyes (n=6). Subconjunctival silicone oil was reported in one eye. Siqueira et al 2007, reported subconjunctival leakage of silicone oil in 9.7% of cases (three out of 31) treated with 23 gauge vitrectomy and silicone oil injection which necessitated second surgery in all three cases to remove silicone oil from the subconjunctival space.^21^ Conversion of one port to 20 gauge was done in one case of retained lens fragments. Retained lens fragments do not represent a contraindication to sutureless vitrectomy. Cortical and small nuclear fragments may be removed by 23-gauge cutter. If large and hard pieces of nucleus are encountered, conversion of one sclerotomy to 20-gauge is all that is required to accommodate the 20-gauge fragmatome.

Comparing the current series with recent reports of Fine et al 2007,^18^ and Tewari et al 2008,^17^ the results were comparable concerning the visual and anatomical improvement. In the current series, contrary to Tewari report, the majority of cases belong to complicated Vitreoretinal pathology as TRD and RRD while straight forward cases (MH and ERM) represent a minority in this series which encourages the use of 23-gauge in complicated Vitreoretinal pathology. Moreover, the current series reported on cases with silicone oil tamponade which represented 20% of cases.

Being retrospective, non comparative and uncontrolled represents limitations of this study.

In conclusion, the 23-gauge transconjunctival sutureless vitrectomy seems effective for management of wide variety of complicated Vitreoretinal pathology with good safety profile. Complications were rare and compared favorably with or lower than previously reported 25-gauge series.^2^ No cases of endophthalmitis, choroidal detachment, or retinal detachment were reported. Careful evaluation of the sclerotomies at the end of the procedure is important especially in cases of silicone oil tamponade. Suturing is considered if there is any silicone leakage to avoid the bad experience of subconjunctival silicone oil.
